# Diabetic Mastopathy as a Radiographically Occult Palpable Breast Mass

**DOI:** 10.1155/2011/162350

**Published:** 2011-11-03

**Authors:** Uma Thanarajasingam, Beiyun Chen, Cindy L. Tortorelli, James W. Jakub, Karthik Ghosh

**Affiliations:** ^1^Department of General Internal Medicine, Mayo Clinic, Rochester, MN 55905, USA; ^2^Department of Laboratory Medicine and Pathology, Mayo Clinic, Rochester, MN 55905, USA; ^3^Department of Radiology, Mayo Clinic, Rochester, MN 55905, USA; ^4^Department of Surgery, Mayo Clinic, Rochester, MN 55905, USA

## Abstract

Diabetic mastopathy is an uncommon, benign disease of the breast that can occur in women with diabetes and clinically mimic breast cancer. We describe a patient with long-standing type 1 diabetes who presented with a palpable breast mass with negative imaging findings on mammography, ultrasonography, and breast MRI. Surgical biopsy and histopathology confirmed diabetic mastopathy. We use this case to highlight the recognition, radiographic features, pathology, and management of this benign breast condition and emphasize that, in diabetic patients, the differential diagnosis of a new breast mass should include diabetic mastopathy.

## 1. Introduction

 Diabetic mastopathy is an uncommon, benign breast condition, seen in diabetic patients and can masquerade as breast cancer. This diagnostic possibility must be borne in mind during the evaluation of a patient with diabetes and a new breast lump. Soler and Khardori [1] were the first to describe an association between diabetes and breast disease that they termed “fibrous disease of the breast”, in a cohort of premenopausal, long-standing Type I diabetics, most of whom had diabetic complications. Diabetic mastopathy is also referred to as sclerosing lymphocytic lobulitis of the breast, lymphocytic mastitis and fibrosis, and diabetic fibrous breast disease. Patients tend to present with a firm, palpable, non-tender, freely movable lump that can be solitary or multiple, uni-or bilateral, and involve any breast quadrant [2]. 

 Since the original report, only a few hundred case reports/case series have been published in the literature. However, given the often nonspecific or absent radiographic and ultrasonographic features of this condition, it can become a diagnostic dilemma for the physician and a source of great anxiety and concern for the patient. We describe a case of diabetic mastopathy in a Type I diabetic woman to highlight the recognition, management, and pathophysiology of this benign breast condition.

## 2. Case Report

A 33-year-old woman with a long-standing history of insulin dependent diabetes complicated with diabetic retinopathy presented to Breast Clinic for evaluation of a new onset, painless, breast lump. She had incidentally discovered a lump in the upper outer quadrant of the left breast 3 months prior that had gradually increased in size in the interim. She denied breast pain, nipple discharge, or overlying skin changes. With regard to breast cancer risk factors, she was nulliparous, menopausal as she had a hysterectomy for dysfunctional uterine bleeding but ovaries were intact, had no previous history of breast biopsies, and was a nonsmoker. She had no personal or family history of breast or ovarian cancer. Her only medication was a NovoLog insulin pump.

Physical exam was remarkable for a dominant 3 × 1 centimeter hard, irregular, movable, painless mass palpable in the left upper outer quadrant and a smaller less defined lesion in the left lower quadrant of the breast. The differential diagnosis included fibroadenoma, fibrocystic change, malignancy (especially lobular carcinoma), PASH (pseudoangiomatous stromal hyperplasia), and diabetic mastopathy. 

**Figure 1:**
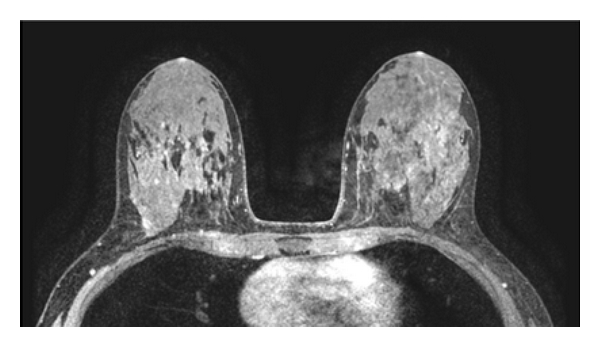
MIP image from breast MRI showing multiple regions of enhancement with no mass in the area of clinical concern left breast.

**Figure 2:**
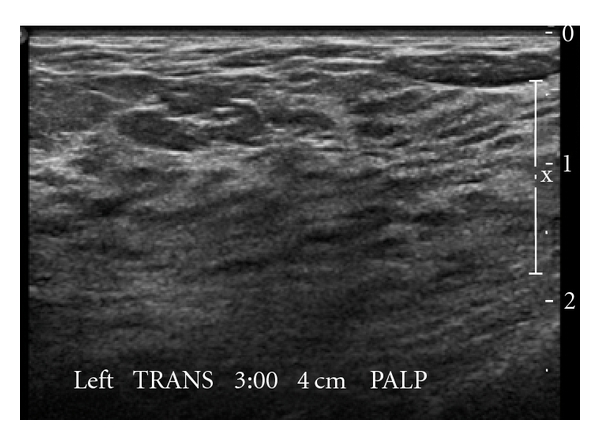
Ultrasound of the palpable abnormality in the left breast shows normal tissue with no mass.

Imaging evaluations included a diagnostic mammogram that revealed extremely dense breasts. Ultrasound exam of the palpable area was also negative for abnormality. A bilateral MRI of the breast was performed (Figure 1) and demonstrated multiple regions of enhancement and a small incidental fibroadenoma of the left breast; no abnormality was detected at the area of palpable concern. A second look ultrasound and left diagnostic mammogram with magnification was obtained, and again while the fibroadenoma seen by MRI was detected by ultrasound this time, the area of the palpable abnormality showed normal tissue with no mass (Figure 2).

**Figure 3:**
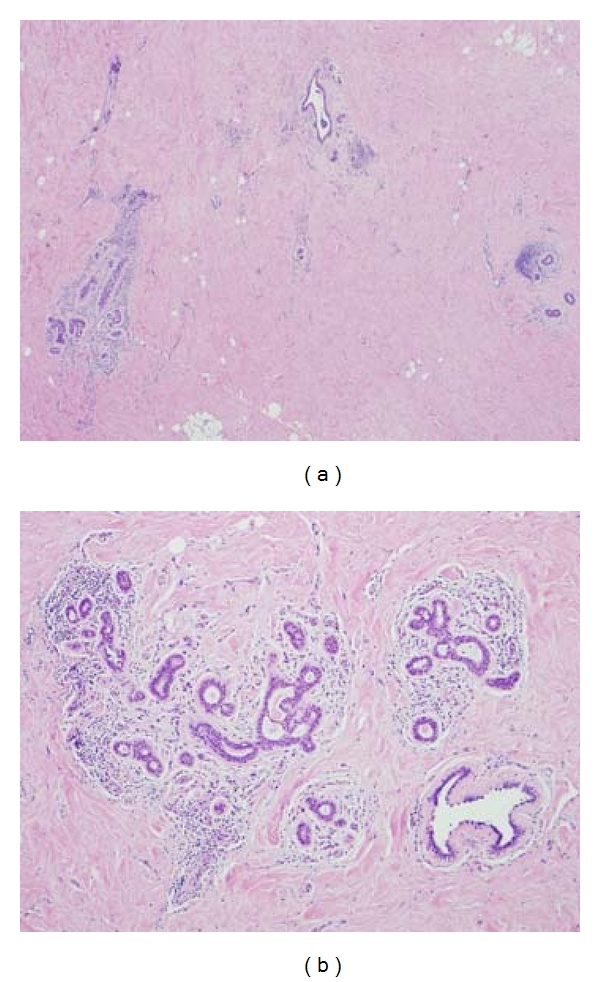
(a) Lymphocytic lobulitis and ductitis with glandular atrophy. Dense fibrosis. (b) Lymphocytic lobulitis, higher magnification.

In view of the size and clinically worrisome features of the palpable, yet radiographically occult mass, an excisional breast biopsy was performed to rule out malignancy. Pathology demonstrated benign breast parenchyma, dense stromal fibrosis, and periductal lymphocyte infiltrate suggestive of diabetic mastopathy (Figures 3(a) and 3(b)).

At her three-month follow-up visit, the patient noted some fullness around her surgical scar that was palpable on clinical breast exam. Diagnostic left breast ultrasound demonstrated a surgical scar, dense breast tissue, but no abnormalities at the area of palpable concern. Continued observation of the breast was recommended with regular clinical follow-up. 

## 3. Discussion

### 3.1. Clinical Features

Diabetic mastopathy typically affects premenopausal Type I diabetic women who manifest complications of diabetes, notably retinopathy and neuropathy [3]. Prior studies report no association between duration of diabetes, glycemic control, and risk for diabetic mastopathy [3]. This is fitting with our patient who had a 23-year history of reasonably well-controlled diabetes complicated by minimal retinopathy. To a much lesser extent, diabetic mastopathy has been reported in men [4] and in patients with Type II diabetes who are often insulin dependent [5, 6]. Clinical features include unilateral or bilateral, single or multiple, nontender, palpable breast masses that are firm to hard in consistency, mimicking malignancy, and mandating further evaluations. 

### 3.2. Imaging

As illustrated by this case, mammography alone, while sensitive for the detection of malignancy, yields no specific features that will result in a diagnosis of diabetic mastopathy. Occasionally, on mammography, regions of asymmetry or ill-defined masses without microcalcifications are associated with the area of palpable concern [7] However, heterogeneously dense breast parenchyma, as noted in our patient, is the most commonly reported pattern seen [7]. Ultrasonographic features of diabetic mastopathy include ill-defined hypoechoic areas with strong acoustic shadowing [8] and lack of color flow on Doppler imaging [7]. MRI findings in diabetic mastopathy have been only sporadically reported and are nonspecific. Patchy parenchymal enhancement has been reported after administration of contrast in patients with diabetic mastopathy, as was seen in our patient [7, 9] In a recent case report, Isomoto et al. suggest that MRI with diffusion-weighted imaging may be helpful in distinguishing diabetic mastopathy from malignancy [10], but this has not yet been validated. 

The aforementioned radiographic findings are nonspecific and cannot rule out malignancy, and biopsy is warranted for a definitive diagnosis. Core needle or excisional biopsy is recommended for tissue diagnosis. Fine needle aspiration biopsy is often nondiagnostic in over 50 percent of cases due to scanty cellularity of the aspirate [11].

### 3.3. Pathology

Several terms have been used in literature to describe diabetic mastopathy including sclerosing lymphocytic lobulitis, ductitis, stromal fibrosis, and perivasculitis [3, 12]. The term diabetic mastopathy was described by Tomaszewski as indicating a constellation of histological features (i) lymphocytic lobulitis and ductitis with glandular atrophy [13], (ii) lymphocytic perivascular inflammation, mostly B cell, (iii) dense fibrosis, and (iv) epithelioid-like fibroblasts [14]. 

### 3.4. Pathophysiology

Prior reports assessing the pathophysiology of diabetic mastopathy have suggested that it is an immune-mediated disorder, supported by the histopathological findings and B-cell predominance [3]. Several theories have been postulated including an inflammatory or immunologic reaction to exogenous insulin [15] or an autoimmune response to advanced glycosylated end products in diabetics [14]. However, the pathophysiology of diabetic mastopathy remains unknown. 

### 3.5. Risk for Cancer and Recurrence

Diabetic mastopathy does not incur an increased risk of breast cancer [3]. The condition is neither malignant nor premalignant [2, 12]. Of note, recurrence of diabetic mastopathy after surgical excision has been reported [2].

### 3.6. Summary of Approach and Follow-Up Recommendations

Diabetic mastopathy is an uncommon, benign disease of the breast predominantly seen in Type I diabetics that can clinically mimic breast cancer. Imaging modalities including mammography, ultrasound, and breast MRI are largely nonspecific and may not definitively rule out malignancy. Tissue diagnosis using core needle or excisional biopsy of the mass is required to establish a diagnosis. Patients and their clinicians need to be informed that this condition can recur and that any new breast lump needs a complete evaluation to rule out malignancy.
